# A protective role for the UPR in galactosaemia

**Published:** 2014-01

Galactosaemia is a rare metabolic disorder characterised by the inability to properly metabolise galactose, a breakdown product of lactose. This leads to toxic accumulation of a precursor metabolite, galactose-1-phosphate (Gal-1-P), which causes liver problems and failure to thrive, as well as long-term developmental consequences if left untreated. The molecular basis of galactose toxicity has remained poorly understood; however, insights are provided in new research from Claudio Masuda and colleagues. Using established yeast models of galactosaemia, they demonstrate that the unfolded protein response (UPR), a cellular response to ER stress, is upregulated in galactosaemic conditions. Upregulation of the UPR is dependent on Gal-1-P synthesis, and impairment of the response makes cells even more vulnerable to cytotoxicity. These results highlight the importance of ER stress in mediating the toxic effects of galactose, and suggest that the UPR plays a protective role that could be harnessed in novel treatments for galactosaemia. Page 55

